# Bioplastic (poly-3-hydroxybutyrate) production by the marine bacterium *Pseudodonghicola xiamenensis* through date syrup valorization and structural assessment of the biopolymer

**DOI:** 10.1038/s41598-020-65858-5

**Published:** 2020-06-01

**Authors:** Yasser S. Mostafa, Sulaiman A. Alrumman, Saad A. Alamri, Kholod A. Otaif, Mohamed S. Mostafa, Abdulkhaleg M. Alfaify

**Affiliations:** 1 Department of Biology, College of Science, King Khalid University, P.O. Box 9004, Abha, 61413 Saudi Arabia; 2 Prince Sultan Bin Abdulaziz Center for Environmental and Tourism Research and Studies, King Khalid University, Abha, Saudi Arabia; 3 Department of Chemistry, Faculty of Science, Jazan University, P.O. Box 114, Jazan, 45142 Saudi Arabia

**Keywords:** Biotechnology, Microbiology

## Abstract

Biobased degradable plastics have received significant attention owing to their potential application as a green alternative to synthetic plastics. A dye-based procedure was used to screen poly-3-hydroxybutyrate (PHB)-producing marine bacteria isolated from the Red Sea, Saudi Arabia. Among the 56 bacterial isolates, *Pseudodonghicola xiamenensis*, identified using 16S rRNA gene analyses, accumulated the highest amount of PHB. The highest PHB production by *P. xiamenensis* was achieved after 96 h of incubation at pH 7.5 and 35 °C in the presence of 4% NaCl, and peptone was the preferred nitrogen source. The use of date syrup at 4% (w/v) resulted in a PHB concentration of 15.54 g/L and a PHB yield of 38.85% of the date syrup, with a productivity rate of 0.162 g/L/h, which could substantially improve the production cost. Structural assessment of the bioplastic by Fourier transform infrared spectroscopy and nuclear magnetic resonance spectroscopy revealed the presence of methyl, hydroxyl, methine, methylene, and ester carbonyl groups in the extracted polymer. The derivative products of butanoic acid estimated by gas chromatography-mass spectrometry [butanoic acid, 2-amino-4-(methylseleno), hexanoic acid, 4-methyl-, methyl ester, and hexanedioic acid, monomethyl ester] confirmed the structure of PHB. The present results are the first report on the production of a bioplastic by *P. xiamenensis*, suggesting that Red Sea habitats are a potential biological reservoir for novel bioplastic-producing bacteria.

## Introduction

Petroleum-based plastic has been rapidly produced in recent decades, and its biodegradation resistance has led to a serious environmental issue for the management of solid wastes^1^. The global demand for bioplastic as a substitute for synthetic plastics has increased due to its nontoxicity, renewability, biocompatibility, and biodegradability^2^. Degradable biobased plastics can be produced from different renewable raw materials (polysaccharides and proteins), plants (starch-based plastics and cellulose-based plastics), and microbial bioplastics (polylactic acid and polyhydroxyalkanoates (PHAs))^3^. PHAs are the most promising type of bioplastic; they are nontoxic, biodegradable and biocompatible and have properties similar to those of conventional plastics^4^. PHAs are biopolymers with diverse structures; as a defense mechanism for surviving stress conditions with nutrient imbalance, PHAs accumulate inside bacterial cells as stored energy^5^. The considerable variations in functional groups from methyl to tridecyl, unsaturated bonds, and chain length make PHAs appropriate biopolymers for many different applications^6^. The most common form of PHAs is poly-3-hydroxybutyrate (PHB), which accumulates in many microbes by binding β-hydroxybutyrate monomers with ester bonds^7^. The universal manufacturing capacity of PHB, which is approximately 30,000 tons/year, is less than 0.1% that of polypropylene, a petrochemical-based plastic; PHB has high annual production rates and will eventually be able to replace polypropylene for various uses^8^. Various technologies use PHB as an interior material for automotive components, electrical devices, sanitary goods, containers, packaging, coating materials, and disposable substances^9^. Meanwhile, in the medical field, antimicrobial materials consisting of PHB nanocomposites and silver nanoparticles have been biosynthesized^10^ and used as drug carriers for wound management and tissue engineering^11^. The structural characterization of intracellular polymers can be performed using UV spectrophotometry, nuclear magnetic resonance (NMR) spectroscopy, Fourier transform infrared (FTIR) spectroscopy, gas chromatography-mass spectrometry (GC-MS), and differential scanning calorimetry^7,12–15^. Economically feasible and sustainable microbial strains, substrates, industrial processes, and extraction methods for PHB are still being developed^4^. The high cellular concentration and production capacity of PHB are responsible for the reduction in the PHB production cost by up to 18%^16^. Extreme stress conditions play an essential role in the biodiversity of marine environments^12,17^. In fact, as an adaptation mechanism, marine microbes produce a variety of metabolites with unique characteristics; thus, they have garnered considerable attention in recent decades as bioresources in biotechnology for the production of many useful materials, such as enzymes, biomass, fine chemicals, and biopolymers^18^. The Red Sea is considered a unique marine ecosystem due to its physical and geochemical properties, and it is one of the most saline marine ecosystems in the world due to various factors, including a low percentage of precipitation, high evaporation, and lack of inflow from major rivers^19^. Furthermore, the Red Sea is characterized as having low nutrient levels, high exposure to UV irradiation, unique coral reef systems, and alkaline properties^20^. These factors have enabled the evolution of microorganisms with unique metabolites^18^. Indeed, the different types of bacterial communities in the Red Sea are reported to be a source of potentially useful bioproducts for biotechnological and pharmaceutical applications, and the gene responsible for PHB synthase exhibits relatively high abundance in the microorganisms living in the Red Sea^19^. The marine bacterial species *Vibrio harveyi*^14^*, Paracoccus* species, *Micrococcus* species^21^*, Erythrobacter aquimaris*^22^, *Halomonas elongata*^23^*, Bacillus megaterium*^24^*, Cupriavidus necator*^25^*, and Haloferax mediterrani*^26^ have been found to be potent producers of bioplastics. Consequently, Red Sea habitats are regarded as a potential source for novel bacterial strains that exhibit efficient PHB production. The cost of the substrates is the major challenge for PHB production; PHB can be produced from many low-cost renewable resources^27–29^. Saudi Arabia is the world’s largest producer of dates, and the number of date palm trees exceeds 25 million; 1.1 million tons of dates and a large amount of date syrup are produced in Saudi Arabia each year and can be used as a relatively inexpensive carbon source for PHB production^12,30^. Therefore, the current study aimed to produce a bioplastic by using novel marine bacteria isolated from the Red Sea, Saudi Arabia; optimized the production conditions and performed a structural assessment of the bioplastic by FTIR spectroscopy, NMR spectroscopy, and GC-MS.

## Results

### Isolation, screening and selection of PHB-producing bacteria

**Figure 1:**
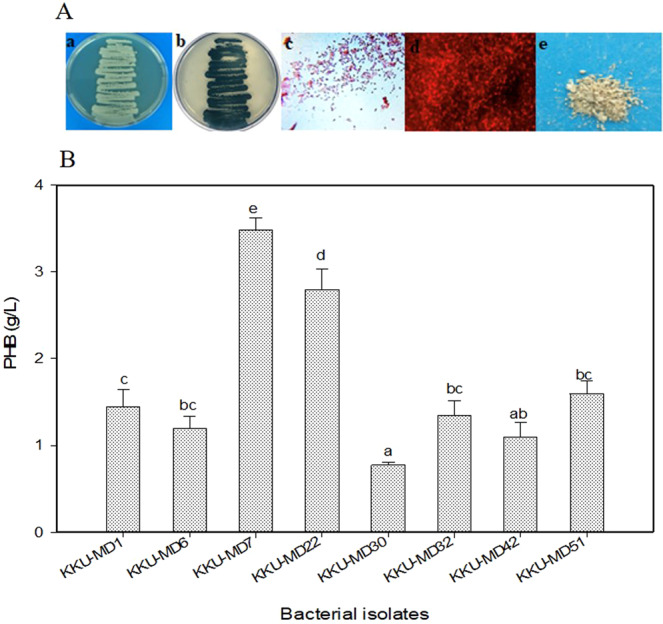
(**A**) The dye-based procedures of PHB granules inside the cells of isolate KKU-MD7: (a) pure culture of the isolate; (b) Sudan Black-B (culture staining); (c) Sudan Black-B (slide staining); (d) Acridine orange (slide staining); (e) Extracted PHB. (**B**) Quantitative screening by submerged fermentation. The means ± standard error (n = 3) are presented. Vertical bars indicate the standard errors of the means. Means followed by different letters are significantly different at P < 0.05 according to Tukey’s HSD test.

A total of 56 marine bacterial isolates were obtained from seawater and sediment samples collected from the Al-Madhaya coast of the Red Sea, Saudi Arabia. The results showed that more bacteria were isolated from the sediment samples (n = 40) than from the seawater samples (n = 16). To determine their ability to accumulate PHB, all bacterial isolates were screened by the rapid detection method of staining in addition to quantitative screening by submerged fermentation. Out of the 56 isolates, 16 dark blue-colored colonies (colony staining) and bacterial cells (slide staining) were observed as good PHB producers (n = 3 from seawater and n = 13 from sediment) using Sudan Black-B staining. Furthermore, the fluorescent dye acridine orange was also employed to confirm the presence of lipid granules of PHB in 16 favorable bacterial isolates. The observation of yellow-colored granules inside bacterial cells showed that KKU-MD7 from seawater (Fig. 1) and KKU-MD22 from sediments had high PHB production capacity. Additionally, six isolates modulated the accumulation of PHB: KKU-MD1 and KKU-MD6 from seawater and KKU-MD30, KKU-MD32, KKU-MD42, and KKU-MD51 from sediments. The eight bacterial isolates that accumulated PHB based on microscopy observations were selected for quantitative screening through submerged fermentation (Fig. 1). The white-to-cream pellet of crude extracted PHB obtained after 72 h at 35 °C was quantified by spectrophotometry. The maximum accumulation of PHB was recorded for bacterial isolate KKU-MD7 (3.49 g/L), followed by KKU-MD22 (2.79 g/L).

### Identification of high PHB-producing bacterial isolate using the 16S rRNA gene and their phylogenetic analysis

**Figure 2:**
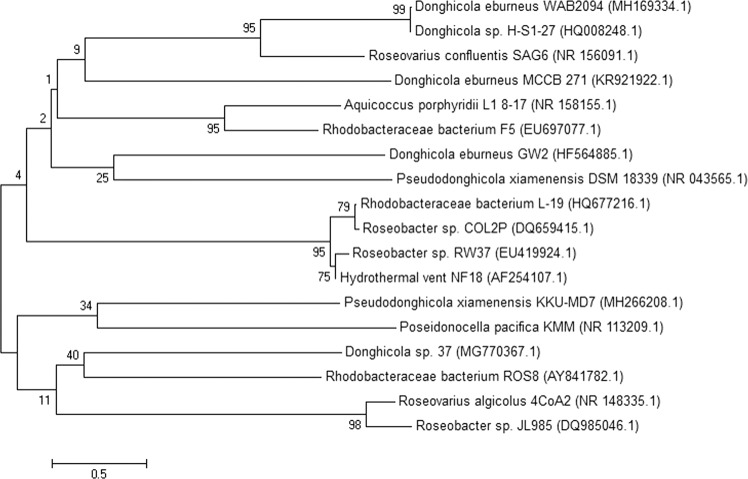
Phylogenetic analysis of the PHB producing bacteria (*P. xiamenensis* KKU-MD7) based on the 16S rRNA sequence constructed through neighbor-joining method using MEGA 5.0 software. The scale bar corresponds to a 0.05 nucleotide substitution per sequence position. The bootstrap values (%) at the nodes. The accession number in GenBank put in parentheses.

The potent PHB-producing isolate KKU-MD7 was subjected to DNA extraction, and its 16S rRNA gene was amplified by PCR. The isolate shared 99.75% identity with *Pseudodonghicola xiamenensis*, as revealed by sequencing and amplification of the 16S rRNA gene. The sequence of the isolate was submitted to GenBank with an explicit accession number (MH266208). A phylogenetic tree of the relationship of the isolate with the other species was constructed (Fig. 2), and contemporary taxonomy for distinguishing between rarely seen bacterial strains was performed for the 16S rRNA gene using molecular techniques. The biosynthesis of the biopolymer by the *P. xiamenensis* isolate was optimized in the following experiments.

### Effect of time on biopolymer production

**Figure 3:**
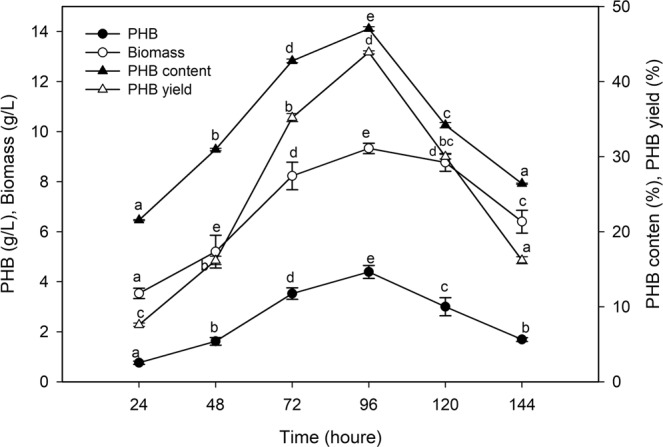
Effect of time course on the biopolymer production by *P. xiamenensis*. Mean ± standard error (n = 3) were presented. Vertical bars indicate the standard errors of the means. Means followed by different letters are significantly different at *P* < 0.05 according to the Tukey’s HSD test.

The fermentation process for PHB production by *P. xiamenensis* had a direct relationship with bacterial growth (Fig. 3). The significant production of PHB (4.39 g/mL) and bacterial biomass (9.33 g/L) increased gradually until 96 h of incubation at the beginning of the stationary phase of growth, corresponding to 46.05% of the PHB content (% of biomass) and 43.9% of the PHB yield (% of the substrate), with a productivity of 0.046 g/L/h. The change in PHB content with growth was initiated after 24 h of incubation (21.53% of biomass), and this value increased gradually to 42.77% of biomass after 48 h and reached a maximum value after 96 h. It was observed that increasing the incubation time to a value above the optimum led to a decrease in PHB production reaching 26.40% of the biomass and 16.9% of the substrate, with a productivity of 0.011 g/L/h after 144 h of fermentation.

### Effect of initial pH on biopolymer production

**Figure 4:**
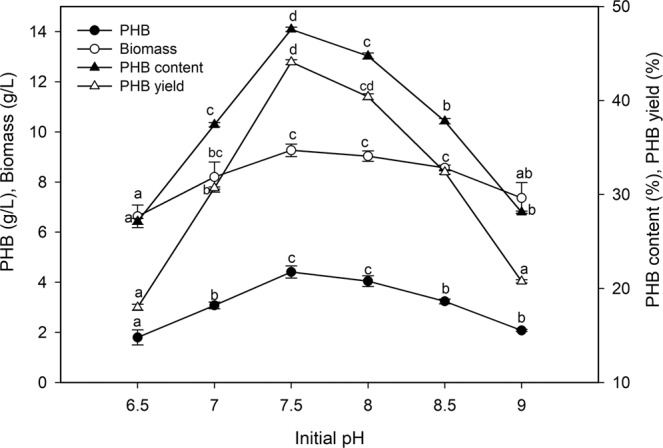
Effect of pH value on the biopolymer production by *P. xiamenensis*. Mean ± standard error (n = 3) were presented. Vertical bars indicate the standard errors of the means. Means followed by different letters are significantly different at *P* < 0.05 according to the Tukey’s HSD test.

To determine the optimum pH for PHB production, the experiment was performed at pH values from 6.5 to 9.0 for 96 h of incubation (Fig. 4). The highest PHB accumulation was achieved at pH 7.5 to 8.0, while the PHB accumulation was dramatically reduced outside this range. The maximum PHB accumulation (4.41 g/L) and bacterial biomass (9.26 g/L) were recorded at pH 7.5, with an increase in PHB content (47.62%), PHB yield (44%) and a productivity of 0.046 g/L/h.

### Effect of temperature on biopolymer production

**Figure 5:**
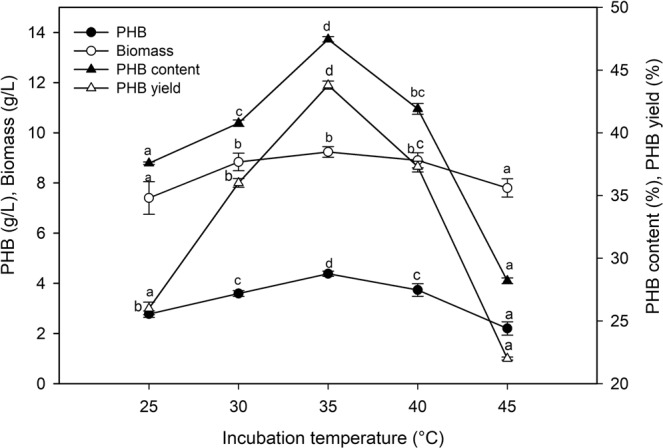
Effect of temperature on the biopolymer production by *P. xiamenensis*. Mean ± standard error (n = 3) were presented. Vertical bars indicate the standard errors of the means. Means followed by different letters are significantly different at *P* < 0.05 according to the Tukey’s HSD test.

To determine the optimal temperature for PHB production, the fermentation process was performed in the range of 25 °C to 45 °C. Bacterial growth and PHB production were strongly influenced by incubation temperature (Fig. 5). The optimum temperature for PHB production (4.38 g/L) and biomass production (9.23 g/L) was 35 °C, which reflected the PHB content (47.45%) and PHB yield (43.8%), with a productivity of 0.046 g/L/h. The fermentation parameters were decreased beyond the optimal temperature value. The PHB concentration (3.73 g/L), biomass (8.90 g/L), PHB content (41.91%), PHB yield (37.3%) and productivity (0.038 g/L/h) were recorded at 40 °C.

### Effect of NaCl concentration on biopolymer production

**Figure 6:**
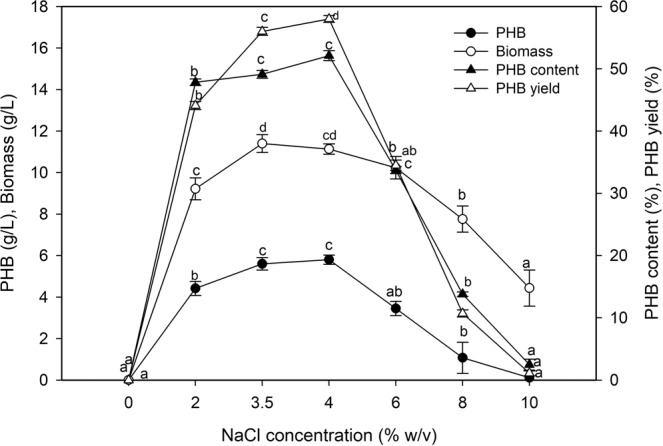
Effect of NaCl on the biopolymer production by *P. xiamenensis*. Mean ± standard error (n = 3) were presented. Vertical bars indicate the standard errors of the means. Means followed by different letters are significantly different at *P* < 0.05 according to the Tukey’s HSD test.

The effect of salt concentration (0% to 10% w/v) on PHB production was investigated. The optimum growth of marine *P. xiamenensis* was observed at 3.5% NaCl, while a slight increase in PHB (5.80 g/mL) was observed at 4% NaCl, resulting in a PHB content of 52.11% and a PHB yield of 58%, with a productivity of 0.060 g/L/h (Fig. 6). The increase in the salt concentration above the optimum value was unsuitable for the fermentation process, where at 6% salt, the bacterial biomass (10.23 g/L), PHB concentration (3.45 g/L), PHB yield (34.5%), and productivity (0.035 g/L/h) decreased.

### Effect of date syrup concentration on biopolymer production

**Figure 7:**
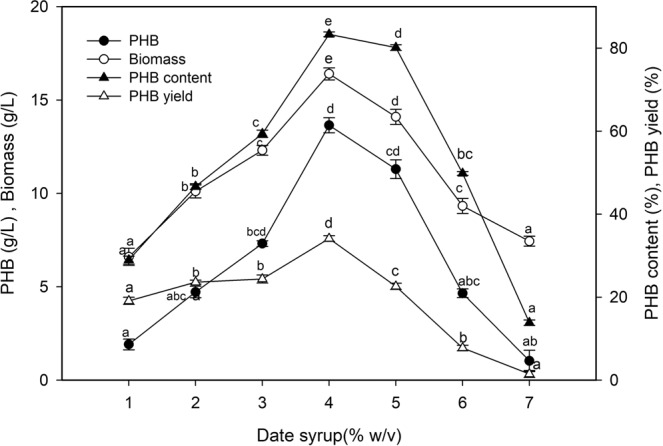
Effect of date syrup concentration as a cheap carbon source on the biopolymer production by *P. xiamenensis*. Mean ± standard error (n = 3) were presented. Vertical bars indicate the standard errors of the means. Means followed by different letters are significantly different at *P* < 0.05 according to the Tukey’s HSD test.

The high cost of refined substrates such as glucose used in PHB production is a major incentive for the development of renewable materials. This experiment was performed to evaluate the efficiency with which *P. xiamenensis* uses date syrup, an important industrial waste in Saudi Arabia. Date syrup contains a high concentration of fermentable sugars (79.5% total sugars, with 42% glucose, 35% fructose, and 7.4% sucrose), indicating its utility as a medium in the fermentation production of PHB. The bacterial biomass (16.40 g/L) and PHB concentration (13.65 g/L) were optimal with 4% (w/v) date syrup, with a 2.36-fold increase in PHB concentration (Fig. 7). The PHB content and productivity increased to 83.23% of the biomass and 0.142 g/L/h, respectively, while the PHB yield (34.12% of the date syrup) decreased, compared with those observed for glucose.

### Effect of nitrogen sources on biopolymer production

**Figure 8:**
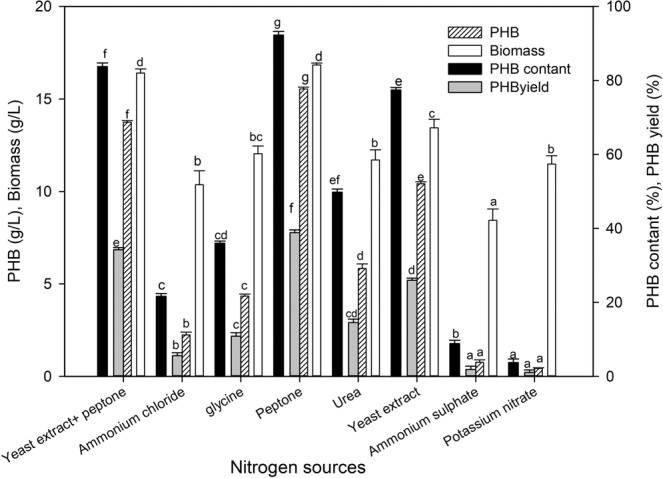
Effect of nitrogen source on the biopolymer production by *P. xiamenensis*. Mean ± standard error (n = 3) were presented. Vertical bars indicate the standard errors of the means. Means followed by different letters are significantly different at *P* < 0.05 according to the Tukey’s HSD test.

The effect of organic (glycine, peptone, urea, yeast extract) and inorganic (ammonium chloride, ammonium sulfate, potassium nitrate) nitrogen sources on PHB production by *P. xiamenensis* was studied. Peptone was the best source of nitrogen that enhanced the accumulation of PHB within bacterial cells (Fig. 8). The maximum production of PHB with peptone was 13.10% higher than that in the initial medium containing a mixture of yeast extract and peptone. In this state, the PHB content (92.28% of biomass), PHB yield (38.85% of date syrup) and PHB productivity (0.162 g/L/h) increased. However, there was a significant reduction in the production of PHB using the inorganic nitrogen sources ammonium chloride, ammonium sulfate, and potassium nitrate, reaching productivities of 0.023, 0.008, and 0.004 g/L/h, respectively.

### Structural characterization of the biopolymer extracted from *P. xiamenensis*

The chemical structure of the obtained biopolymer was investigated using several analytical procedures.

### FTIR analysis

**Figure 9:**
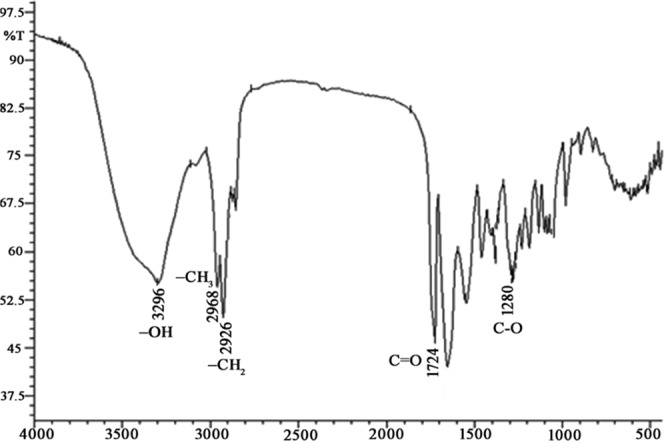
FTIR spectrum of PHB extracted from *P. xiamenensis*. The absorption bands at 3440, 2960, 2932, 1724, and 1280 cm^−1^ correspond to the –OH, –CH_3_, –CH_2_, C = O, and C–O groups.

The functional groups existing in the polymer extracted from *P. xiamenensis* were detected, and FTIR spectral data showed the following absorption bands: (a) a band at 3296 cm^−1^, corresponding to stretching of a terminal OH group; (b) bands at 2968 cm^−1^ and 2926 cm^−1^, indicating C-H stretching in methyl and methylene groups; (c) a band at 1724 cm^−1^, indicative of C = O stretching of an ester group; and (d) a C-O band at 1280 cm^−1^. These absorption bands correspond to the peaks obtained for the standard PHB and confirm the structure of PHB isolated from *P. xiamenensis* (Fig. 9).

### NMR analysis

**Figure 10:**
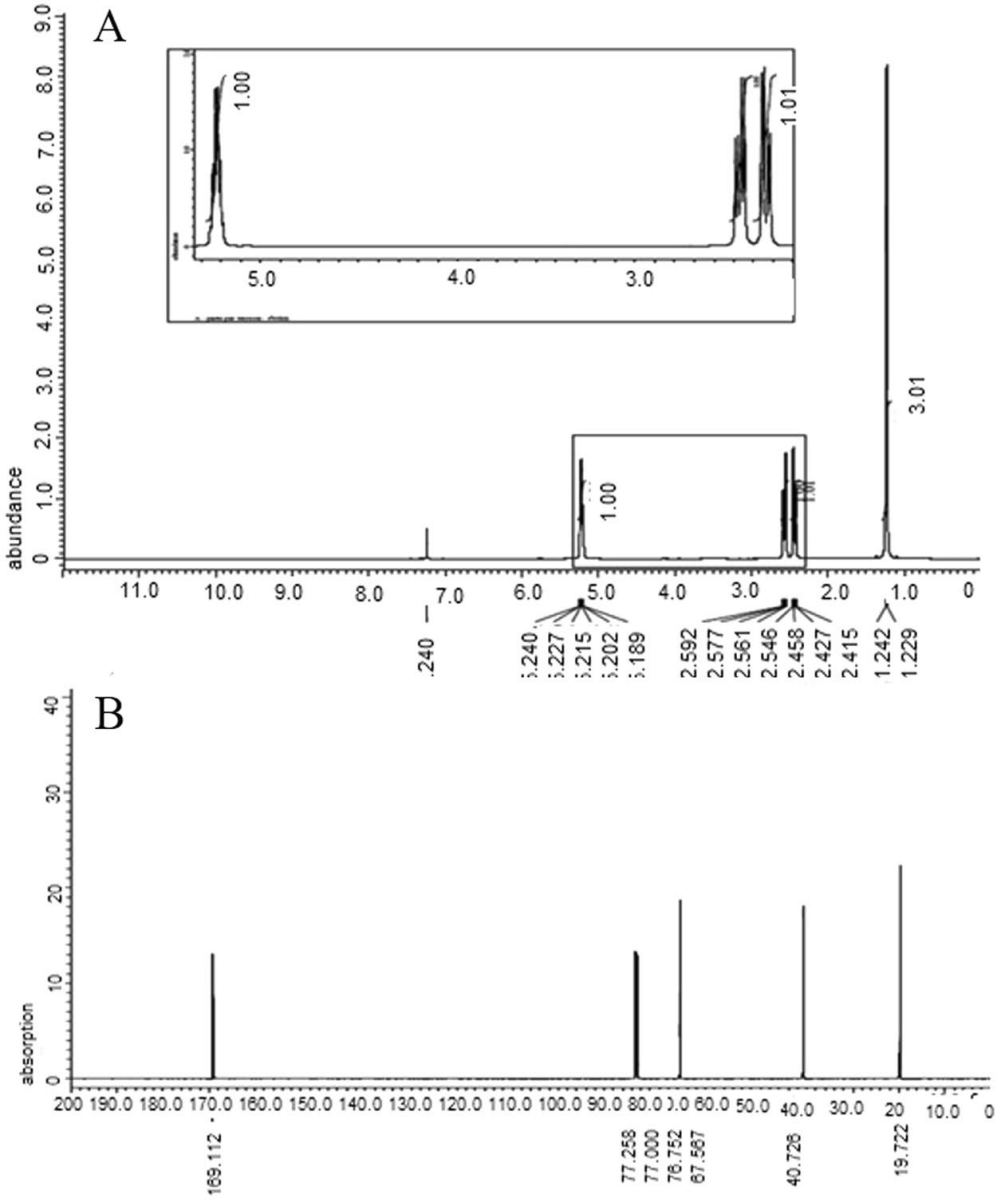
NMR spectrum of PHB extracted from *P. xiamenensis*. (**A**) ^1^H NMR spectrum shows signals at chemical shift δ 1.22–1.23 ppm (d, CH3,), 2.41–2.54 ppm (q, CH_2_,), and 5.18–5.25 ppm (m, CH,). (**B**) ^13^C NMR spectrum shows signals at 169.11, 67.56, 40.72, and 19.58 ppm for the carbon atom of the CO, CH, CH_2_, and CH_3_ groups.

The ^1^H NMR spectrum of the extracted PHB revealed signals of a methyl group doublet at a chemical shift of δ = 1.22 ppm. The pair of quadruplets of a methylene group joint to a carbonyl group (-CH_2_) was observed at δ = 2.41–2.54 ppm. Multiple signals appearing at δ = 5.18–5.25 ppm corresponded to a methine group (-CH). As shown in Fig. (10A), the obtained signals for ^1^H NMR can be described as follows: δ 1.25–1.23 ppm (d, CH_3_, J = 6.4 Hz), 2.6−2.4 ppm (DQ, CH_2_, J = 7.6, 5.6 Hz) and 5.24-5.21 ppm (m, CH, J = 5.6 Hz). The ^13^C NMR spectrum was analyzed to confirm the structure of the extracted biopolymer. The signal of the quaternary carbon of the carbonyl group was detected at 169.11 ppm, while the signal of the CH group was observed at 67.56 ppm. At a high value of 40.72 ppm, the signal for the CH_2_ group appeared close to the carboxyl group, whereas the signal of the carbon of the methyl group was observed at 19.58 ppm (Fig. 10B).

### GC-MS analysis

**Figure 11:**
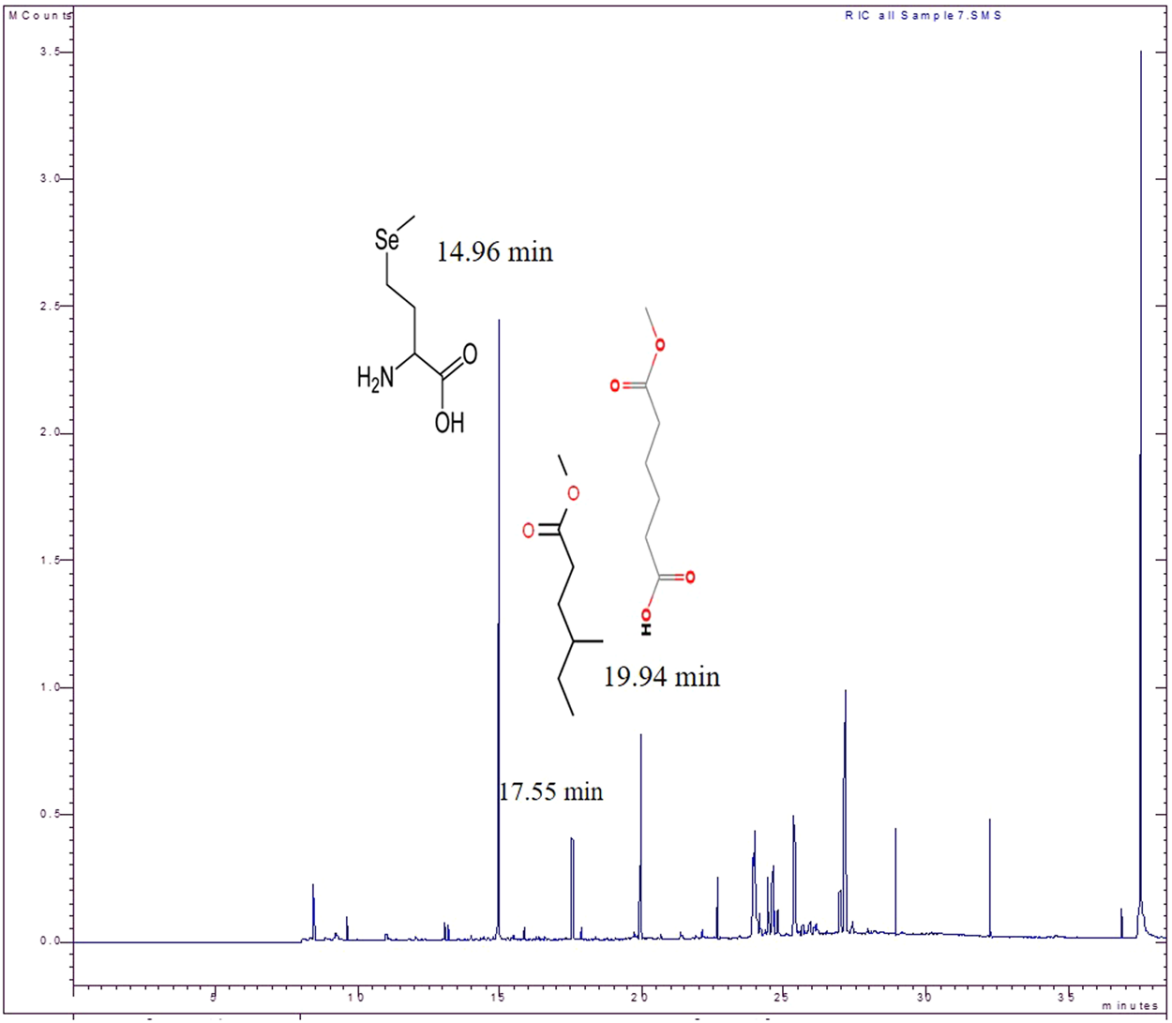
GC-MS chromatogram of PHB extracted from *P. xiamenensis*. The chromatogram shows three monomeric compositions of PHB; Butanoic acid 2- amino-4-(methylseleno)-, (s); Hexanoic acid, 4-methyl-, methyl ester; and Hexanedioic acid, monomethyl ester.

The GC-MS spectra of methanolyzed PHB extracted from *P. xiamenensis* are shown in Fig. (11); three prominent peaks with retention time (Rt) values of 14.96, 17.55 and 19.94 min were observed. These peaks correspond to multiple butenoic acid derivatives, butanoic acid, 2-amino-4-(methylseleno), hexanoic acid, 4-methyl-, methyl ester, hexanedioic acid, and monomethyl ester, confirming the presence of PHB.

## Discussion

In the present study, marine bacterial strains were isolated from the Al-Madhaya coast of the Red Sea in the southern region of Saudi Arabia. The PHB-producing *Erythrobacter aquimaris* was isolated from the mangrove rhizosphere of this site^22^. Fewer isolates were obtained from the seawater samples than from the sediment samples, probably because the prevalent environmental conditions and rainy season diluted the water^31,32^. Intracellular PHB granules were directly detected in marine bacteria by a dye-based procedure, and black- and yellow-colored lipid granules were considered indicators for PHB accumulation by Sudan Black-B and acridine orange staining, respectively^31,33^. The high susceptibility of intracellular lipid granules to staining with Sudan Black-B and the fluorescent dye acridine orange was explained by incorporation with the structural components of the cells^34^ and the ease of distinguishing between the cell contents at different wavelengths, respectively^35^. The current investigation demonstrates that the habitats of the Red Sea were a potential source for bacterial isolates producing PHB. The Red Sea ecosystem promoted the accumulation of PHB by microbial communities as a survival mechanism due to low sediment and nutrient levels as a result of the absence of river inflow^19^.

The molecular identification of bacteria by the 16S rRNA gene is an accurate modern method that has replaced the traditional methods that rely on phenotypic identification^33,36^. 16S rRNA is a ribosome component of the 30S subunit in prokaryotes. It is encoded by the 16S rDNA gene, which is considered the most common genetic marker^36^. The most active isolate exhibiting the accumulation of PHB granules, KKU-MD7, was identified based on the 16S RNA gene as *P. xiamenensis*. This species is a slightly halophilic bacterium belonging to the phylum Proteobacteria, a predominant bacterial phylum in marine habitats^37^. Marine ecosystems such as the Red Sea^13,22^ promote diverse biopolymer-producing microbes, which can synthesize unique genes and enzymes^17^. The marine bacteria producing PHB that were identified as *Ralstonia*, *Pseudomonas* sp., *Bacillus* sp., and *Vibrio proteolyticus* based on 16S rRNA were isolated from marine environments^15^. Several new bacterial strains producing high amounts of PHB were isolated from various marine environments and have been identified by analyzing 16S rRNA sequences, including bacteria from the mangrove rhizosphere (*Bacillus thuringiensis, Erythrobacter aquimaris*)^13,22^, sediments (*Vibrio* sp.)^5,38^, surface seawater (*Alteromonas lipolytica*)^39^, the tunicate *Phallusia nigra* (*V. harveyi* MCCB 284)^14^, and sponges (*Bacillus megaterium*)^24^ The variation in microbial communities in marine habitats may be affected by pollution, as seawater can become rich in organic and inorganic nutrients. The bacteria producing PHB identified based on the 16S rRNA sequence, namely, *Bacillus*, *Staphylococcus*, *Paracoccus*, and *Micrococcus*, were found at a polluted site, while *Rhodococcus* and *Methylobacterium* were found at a nonpolluted site^21^.

*P. xiamenensis* was able to accumulate PHB within 96 h of incubation, and the compatibility of PHB accumulation and biomass with PHB yield and productivity was in accordance with the results previously reported for PHB production by *Bacillus subtilis* (54.1%) and *E. coli* (47.16%)^40^. Various optimal fermentation periods have been mentioned for the production of PHB at the beginning of the stationary phase of growth depending on bacterial strains. Some reports refer to 48 h of incubation as being optimal for PHB production by *Bacillus pasteurii* and *Micrococcus luteus*, affording maximum PHB yields of 36.41% and 34.59%, respectively^41^, while for *Rhizobium etli* E1 and *Pseudomonas stutzeri* E114, PHB yields of 81.80% and 83.02%, respectively, were achieved^42^. In addition, *Bacillus* spp. accumulated 55.6, 51.6, 37.4 and 25% PHB using sugar cane bagasse, corn cob, teff straw, and banana peel as carbon sources^43^. Moreover, marine *Saccharococcus thermophilus*^33^ and *V. harveyi*^14^ had maximum PHB yields of 0.701 g/L and 1·2 g/L based on dry cell weight, respectively, after 72 h. Additionally, high PHB production (7.3 g/L) was achieved at 120 h by *E. aquimaris*^22^. Extending the fermentation period reduced PHB production, which can be explained by an explicit positive relation between PHB production and bacterial biomass, where both decreased as carbon was consumed, resulting in the exhaustion of PHB^31,42^ in addition to decaying enzyme activity for PHB biosynthesis^40^. Clearly, the microbes accumulate PHB as a storage carbon, and their survival is promoted by the hydrolysis of PHB by PHB depolymerase, as reported for *Rhodovulum sulfidophilum* P5 and *V. harveyi*^14,31^.

It is crucial to consider the bioavailability of trace minerals^40^ and the activity of the PHB synthesis enzyme PHA polymerase^31^ by monitoring the initial pH of the fermentation medium. Optimum PHB production by *P. xiamenensis* was achieved at pH 7.5, while acidic and highly alkaline pH values were not suitable for PHB production. The optimum growth of *P. xiamenensis* isolated from the seawater of the Taiwan Strait, China occurred at about pH 7.0^44,45^. Although the estimated pH in the marine samples collected from the Al-Madhaya coast of the Red Sea, was 8.2, the bacterial growth and PHB production were significant at pH values from 7 to 8.5, which may be explained by bacterial adaptation to adverse conditions^46^. The results obtained were highly comparable with the data for marine *Bacillus megaterium* MSBN04, and high PHB production (56.81%) with the maximum PHB yield (8.637 mg/ g of the substrate) was achieved at pH 7.5 and drastically reduced at acidic pH compared to alkaline pH^24^. The production of PHB was supported at pH 7 to 9 by the marine bacteria *Vibrio azureus*^17^, *B. pasteurii* and *M. luteus*^41^. Additionally, the highest PHB production (3.2–6.8 g/L) was observed at pH 7.0–7.5 for *R. etli*, *P. stutzeri*, and *Bacillus* sp^42,43^. On the other hand, pH 8.0 was found to be optimal for PHB accumulation by *E. aquimaris*^22^*, V. harveyi*^14^, and *Azotobacter chroococcum*^33^.

The effect of incubation temperature on the changes in bacterial growth and PHB production could be due to the effects on the enzymes involved in PHB synthesis^43^. The optimum PHB production and biomass were obtained at 35 °C, as mentioned previously^17,22,40^, and the evaluation parameters for PHB production were highly comparable. The PHB production by marine *V. azureus* reached 0.48 g/L, and the PHA content reached 0.426 g/g of cell dry weight^17^. Furthermore, the marine *Cupriavidus taiwanensis*^47^ and *B. subtilis*^48^ MSBN17 were able to accumulate high levels of PHB (7.0–19.0 g/L) at 30 °C, while in the same temperature range, marine *V. harveyi* MCCB 284 showed a PHB yield of 2.3 g/L^14^. Additionally, the production of PHB by *S. thermophilus* (0.701 g/L) was optimal at 50 °C with molasses^33^.

The salt concentration is a pivotal factor for marine bacterial growth and biopolymer production^17^. There was no growth in the absence of salt (0%), which confirms that the strain was related to obligate halophiles^44,45^. The *P. xiamenensis* strain exhibited considerable adaptability at various salt concentrations, similar to that of seawater (3.5–4%). The highest bacterial biomass was obtained at 3.5% NaCl compared with 4%, which was optimal for PHB production (5.80 g/L), PHB content (52.11%), and PHB yield (58%) associated with the stress response. These findings may be explained by the accumulation of a high amount of PHB as a survival mechanism depending on bacterial adaptation of the metabolic pathway for osmotic pressure equilibration^49^. Various studies have referred to the stimulation of PHB production as a response to high osmotic pressure for *V. proteolyticus*^15^, *H. elongata* 2FF^23^, *C. necator*^25^, and *H. mediterranei*^26^. Increasing the salt concentration to higher than the optimal value decreased bacterial growth and therefore PHB accumulation due to the effect of high osmotic stress^14^. These results reflected the necessity of monitoring the salinity of the medium to an appropriate extent to prevent high osmotic stress and its effect on growth and PHB production. Various salt concentrations were reported as being optimal for PHB based on the bacterial strains used, such as 1.5% (*V. azureus*)^17^, 2% (*V. harveyi*)^14^, 3% (*B. subtilis*)^50^, 5% (*V. proteolyticus*)^15^, and 10% (*H. elongata*)^23^.

The carbon source is a major cost-associated factor in the production of PHB^27,30^. The bacterial behavior for utilizing various renewable resources as complex carbon sources varies depending on the nature of the materials and the enzymes synthesized by the microbes^42^. Investigations regarding the production of bioplastic from date syrup as one of the most abundant and inexpensive byproducts in Saudi Arabia are limited despite the sugar content of date syrup exhibiting potential uses in the fermentation process^12,29^. The use of date syrup at 4% (w/v) resulted in higher bacterial biomass (16.40 g/L) and PHB production (13.65 g/L) than that obtained using glucose, reflecting the associated PHB content (83.23% of the biomass), PHB yield (34.12% of the date syrup) and productivity (0.142 g/L/h). This is probably due to the complexity and numerous organic nitrogen compounds present in date syrup^29^. These values were higher than the corresponding values reported in the literature. High PHB production (3.6 g/L) and biomass (5.1 g/L) of *Bacillus* sp. were recorded using 5% date syrup^51^, while maximum PHB production (5 mg/ml) by *Bacillus* sp. was observed with 8% date syrup^12^. Moreover, PHB production by *B. megaterium* using date syrup and beet molasses was high, reaching 50–52% PHB^29^. In addition, the high PHB production by *Pseudomonas* sp. (15–20 g/L) obtained using rice bran, dates, and soy molasses corresponded to a PHB content of 90.9%, 82.6%, and 91.6%, respectively^30^. Other renewable resources were also used for PHB production, such as waste paper hydrolysate by *Burkholderia sacchari*^27^, affording the highest PHB production (1.6 g/L) and PHB content (44.2%), with a PHB yield of 15% and productivity of 0.033 g/L/h. Additionally, kitchen waste-derived organic acids were used as a carbon source for PHB production by *C. necator*, affording a PHB content of 52.79% with a PHB yield and productivity of 0.38 g/g and 0.065 g/L/h, respectively; the productivity increased in fed-batch culture to 0.242 g/L/h^28^.

Different organic and inorganic nitrogen sources affect both bacterial biomass and PHB production, where the strains accumulate lipid granules under unbalanced nutritional conditions as a survival mechanism when nitrogen is depleted^52^. The PHB productivity (0.162 g/L/h) and PHB yield (38.85%) obtained using peptone as the optimal nitrogen source for PHB accumulation (15.54 g/L) were superior, possibly because it is considered a complex nitrogenous source and the precursor for amino acids and growth factors^32^. The values were higher than those obtained by *E. aquimaris* (7.3 g/L)^22^ and *Bacillus* spp. (5.2 g/L)^43^. The mixture of yeast extract and peptone in the initial medium was not suitable for PHB production^4,33^. Nevertheless, the prospect of there being more than one mechanism for PHB accumulation among the various microbes is being considered; ammonium sulfate was optimal for PHB production by *B. sacchari*^27^, *R. etli* E1 and *P. stutzeri*^42^.

The chemical structure of the extracted biopolymer was assessed using several analytical procedures^7,12,13^. The spectra obtained for the extracted PHB displayed a high correspondence with that for pure PHB as well as with previously reported spectra^14,15^. In the present study, the values of the FTIR absorption bands of the carbonyl group, -OH group, -C-H group, and -CH_2_ and -CH_3_ groups were related to values reported in recent studies^53–56^. One of the most common chemical groups in all types of PHAs is the carbonyl group (C = O)^41,43^. The strong band at 1724 cm^−1^ for carbonyl stretching of the ester group of the extracted PHB is analogous to that for the standard PHB and the peak of the C = O group for PHB from *Bacillus cereus*^31,43^. FTIR spectra of PHB extracted from *H. elongata*^23^ and *S. thermophilus*^33^ showed C = O stretching vibrations at 1721 cm^−1^ and 1726 cm^−1^, respectively. The FTIR spectra of the PHB extracted from *B. sacchari* showed strong bands for the carbonyl group of the ester (1720.39 cm^−1^), the band for methyl groups (1379.57 cm^−1^), and the bands for the stretching vibration of methylene and methane groups (1277.68 cm^−1^ and 2930.54 cm^−1^)^27^. The peaks at 2960 and 2968 cm^−1^ for the polymer extracted from *P. xiamenensis* corresponded to C-H stretching methyl and methylene groups, respectively, which was consistent with previous findings^50^. The peaks at 3406 cm^−1^ showed strong H bond stretching by the terminal OH groups^43^. The spectrum of extracted PHB also showed a band of the C-O stretch corresponding to the C–O group identified at 1278 cm^−1^ for PHB from *H. elongata*^23^. Additionally, the bands at 1000–1320 cm^−1^ were distinctive for the C–O group of the esters^31^. FTIR spectroscopy of PHB extracted from *Pseudomonas* sp. Strain‐P showed various absorption bands at 3456.43 cm^−1^ (OH group), 2986.44 (‐CH3 group), 2858.50 cm^−1^ (‐CH_2_ groups), 1637.56 (C = O), and 1261.44 cm^−1^ for the C–O group^30^. Other prominent bands were also observed by FTIR spectroscopy, which may be due to interactions between the OH and C = O groups resulting in a stretching shift^57^.

NMR spectroscopy is a main procedure for efficiently detecting biopolymer structures^58^. The ^1^H NMR spectrum of PHB extracted from *P. xiamenensis* showed absorption bands for methyl, methylene and methane groups, confirming that the polymer structure was PHB. Based on ^1^H NMR spectroscopy, the doublet peaks at 1.3 ppm (methyl group), the doublet of the quadruplet peaks at 2.46 ppm (methylene group), and the multiplex peaks at 5.24 ppm (methane group) were assigned for the PHB structure of *B. sacchari*^27^. Additionally, three signals of various groups, namely, methyl (1.21 ppm), methylene (2.56 ppm), and methine (5.22 ppm), were reported in the ^1^H NMR spectrum of PHB produced by marine *V. harveyi*^14^. Furthermore, the absorption values for methyl, methylene and methane groups with peaks of 1.23, 2.5 ppm and 5.2 ppm, respectively, were recorded for PHB produced by *B. subtilis* MSBN17 and *B. cereus*^50,59^.

The ^13^C NMR spectrum of the extracted polymer was confirmed based on the shift in the signal of the carbonyl, methane, methylene, and methyl carbon groups. The ^13^C NMR spectrum of the PHB structure extracted from *B. sacchari* showed various groups: carbonyl, methane, methylene and methyl groups^27^. The ^13^C NMR spectrum of the PHB produced by *V. harveyi*^14^ and *Lysinibacillus sphaericus*^58^ showed chemical shift signals corresponding to the carbonyl (169.14, 169.143 ppm), methane (67.61, 67.656 ppm), methylene (40.79, 40.864 ppm), and methyl (19.76, 19.787 ppm) groups.

The GC-MS method was applied to detect the monomeric components of extracted PHB^7^. Three distinct peaks identical to the peaks of various derivatives of butenoic acid were revealed for PHB extracted from *P. xiamenensis* [butanoic acid, 2-amino-4-(methylseleno); hexanoic acid, 4-methyl-, methyl ester and hexanedioic acid, monomethyl ester], corroborating the existence of PHB. The GC-MS spectra of PHB extracted from *Bacillus licheniformis* MSBN1 and *B. megaterium* revealed major peaks resembling methyl 3-hydroxybutyrate, indicating the polymer structure^50,60^. Additionally, the structure of PHB extracted from *B. cereus* was also assessed based on various peaks in the GC-MS spectrum corresponding to various derivatives of butenoic acid^59^.

## Materials and Methods

### Marine samples

Seawater and sediment samples were collected in a sterilized glass screw-cap bottle; the samples were collected from the Al-Madhaya coast of the Red Sea, Saudi Arabia, at a depth of 20 cm. The temperature (34 °C), pH (8.2), and salinity (35.2 ppt) of the marine samples were assayed using portable meters (OAKTON).

### Date syrup preparation

Date syrup was prepared from date fruits (Khalas) collected from Abha city, Saudi Arabia. The date syrup was prepared by blending the water and dates (1:5 ratio), heating at 70 °C for 2 h, and then centrifuging at 6000 rpm for 10 min. The characteristics of the date syrup were estimated using HPLC (Agilent Technologies, 1200 Model Infinity, USA)^12^.

### Isolation of PHB-producing marine bacteria

The marine samples were serially diluted, and 0.1 mL of each dilution was spread on Zobell marine agar with 1% glucose as a carbon source^61^. The cultures were incubated at 35 °C for 72 h at 150 rpm in an incubator shaker (Shell Lab, USA), and the purified colonies were preserved at 4 °C on slants of the same medium.

### Screening of PHB-producing marine bacteria

Sudan Black-B (0.05%) was used for staining the culture plates for 30 min. A bacterial smear was stained with Sudan Black-B (0.3%, w/v) for 20 min and then immersed in xylene before being stained with safranin. Additionally, 0.05 mL of the fluorescent dye acridine orange was added to 0.01 mL of a 72-h-old bacterial culture and incubated at 35 °C for 30 min. After centrifugation of the mixture (5 min at 6000 rpm), the smear was prepared and examined by a fluorescence microscope (Nikon, Eclipse-e400, Japan)^33^. The isolates producing PHB at high levels were screened by submerged fermentation in a 250 mL Erlenmeyer flask containing 50 mL of Zobell marine broth supplemented with 1% glucose. The fermentation was carried out for 72 h in an incubator shaker (at 35 °C/150 rpm) with 2% of a 24-h-old culture (1 × 10^7^ CFU/mL). The culture was centrifuged at 6000 rpm for 10 min, and the PHB was extracted and estimated.

### Extraction and analysis of PHB content

The dried cells were washed with acetone and ethanol for 20 min and treated with 50 mL of sodium hypochlorite (30%) and 50 mL of chloroform. This mixture was incubated for 1 h at 37 °C in a shaker at 150 rpm and then centrifuged (5000 rpm for 30 min); centrifugation was performed to remove the cell residues. Pure PHB was obtained by evaporating the chloroform at 40 °C^62^. The white extracted polymer was dissolved in concentrated sulfuric acid at 100 °C for 10 min to convert it into brown-colored crotonic acid, which was assayed at 235 nm (PerkinElmer Lambda, UV/VIS spectrophotometer, USA)^7^. Standard solutions of pure PHB were prepared at concentrations ranging from 20 to 200 µg/mL.

### Identification of isolated bacteria (KKU-MD7) by 16S rRNA gene sequencing

The bacterial genomic DNA of the isolate exhibiting high levels of PHB production was extracted using a QIAamp DNA Mini Kit (Qiagen Inc., USA). This DNA was used as a template for amplification of the 16S rRNA gene using universal primers (5′ CCA GCA GCC GCG GTA ATA CG 3′ and 5 ′ATC GG(C/T) TAC CTT GTT ACG ACT TC 3′)^63^. An electrophoresis unit was used to run the gel for the migration of the amplified genes, and the PCR product was compared against a 1-kb DNA ladder to verify the presence of properly sized amplicons. A DNA Purification Kit (version 2.0, Qiagen Inc., Valencia, CA, USA) was used to purify the product of the right size, which was then sequenced (Macrogen, Korea). The obtained sequence was aligned and compared with the sequences deposited in GenBank. The phylogenetic tree was constructed with MEGA, version 5.10, using a neighbor-joining algorithm.

### Enhancement of PHB production

PHB production was carried out in a 250 mL Erlenmeyer flask containing 50 mL of Zobell marine broth medium, and the flasks were incubated in a shaker at 150 rpm. To enhance PHB production, the time (12–144 h); pH value (6.5–9.0); temperature (25–45 °C); salt concentration (2–10% w/v); concentration of date syrup (1–7% w/v), as an inexpensive carbon source; and nitrogen source (organic sources: peptone, glycine, urea, yeast extract; inorganic sources: ammonium chloride, potassium nitrate, and ammonium sulfate) were optimized. The biomass (cell dry weight, g/L) was monitored after centrifugation of the culture at 8000 rpm for 20 min and drying until constant weight at 70 °C. The optimized experiments were conducted in triplicate. The productivity of PHB (g/L/h) = concentration of PHB (g/L)/fermentation time (h). The PHB content (%, w/w of the dry biomass) = (weight of PHB/dry cell weight) × 100. The efficiency of the conversion efficiency to obtain PHB from the substrate expressed as the PHB yield (%, w/w of the substrate) was calculated as the concentration of PHB produced (g/L) × 100/substrate concentration (g/L).

### Structural assessment of extracted PHB

**FTIR**^61^: The mixture of extracted PHB and chloroform was placed on a KBr disk, and the functional groups were analyzed using an IR spectrophotometer (Shimadzu, Japan) at wavelengths ranging from 4000 to 400 cm^−1^. **NMR**^14^: Both ^1^H NMR and ^13^C NMR spectra were obtained to show the chemical structure of the polymer using an NMR 500 MHz Ultra Shield Bruker spectrophotometer (San Jose, USA) and an ECA-500 II Jeol spectrophotometer (JEOL Ltd., Japan). **GC-MS**^59^: The organic phase of methanolysis of PHB containing the resulting methyl ester monomers was assayed by a Varian Saturn 2100 T GC-MS equipped with a Saturn 2100 mass detector (Mundelein, USA), and the mass spectra were analyzed by NIST 98 mass library software (USA).

### Statistical analysis

One-way analysis of variance (ANOVA) was used, and the significant differences between the means (n = 3) were identified using Tukey´s HSD test at *p* ≤ 0.05. All statistical analyses were performed using Statistica software 7.1.

## Conclusion

The current investigation focused on the production of the bioplastic PHB using a potent novel marine bacterium, *P. xiamenensis*, isolated from the Red Sea, Saudi Arabia. The maximum PHB production of 15.54 g/L was observed after optimizing the fermentation conditions: time (96 h), pH (7.5), temperature (35 °C), NaCl (4% w/v), and peptone as nitrogen source. Low-cost date syrup (4% w/v) afforded a PHB content of 92.2% (w/w of the biomass) and PHB yield of 38.85% (w/w of the date syrup) with a productivity rate of 0.162 g/L/h, leading to substantially improved production costs. Structural assessment of the bioplastic by FTIR spectroscopy, NMR spectroscopy, and GC-MS showed the hydroxyl, methyl, methylene, methine, and ester carbonyl groups in addition to the derivative of butanoic acid, indicating that the polymer was PHB based on the structure. These findings are the first report on the production of a bioplastic by *P. xiamenensis* using date syrup as an inexpensive substrate. Further investigations will be necessary to increase the PHB productivity of *P. xiamenensis* using fermentation processes and modeling studies.
